# *CHI3L1* polymorphisms associate with asthma in a Taiwanese population

**DOI:** 10.1186/1471-2350-15-86

**Published:** 2014-07-23

**Authors:** Yishan Tsai, Yingchin Ko, Mingshyan Huang, Mengchih Lin, Chaochien Wu, Chinchou Wang, Yunxuan Chen, Jianing Li, Yuting Tseng, Tsunai Wang

**Affiliations:** 1 Department of Public Health, College of Health Science, Kaohsiung Medical University, No. 100, Shi-Chuan 1st Rd, Kaohsiung 807, Taiwan; 2 Environment-Omics-Diseases Research Center, China Medical University Hospital, Taichung, Taiwan; 3 Graduate Institute of Clinical Medical Science, China Medical University, Taichung, Taiwan; 4 Division of Pulmonary and Critical care Medicine and Geriatric Medicine, Department of Internal Medicine, Kaohsiung Medical University Hospital, College of Medicine, Kaohsiung Medical University, Kaohsiung, Taiwan; 5 Division of Pulmonary and Critical Care Medicine, Chang Gung Memorial Hospital and Chang Gung University College of Medicine, Kaohsiung, Taiwan

**Keywords:** Asthma severity, CHI3L1, Lung function, Polymorphism, YKL-40

## Abstract

**Background:**

A genome-wide association study uncovered *Chitinase 3 like 1* (*CHI3L1*) as a candidate gene for asthma susceptibility. *CHI3L1*, which encodes the YKL-40 protein, is associated with asthma in Western European and American populations and with atopy in a Korean population. However, asthma-associated polymorphisms remain unknown for a Taiwanese population.

**Methods:**

We enrolled 628 adult asthmatic patients and 1:1 age-sex matched community-based controls in southern Taiwan and performed a combined effect sizes analysis to test if *CHI3L1* polymorphisms were related to genetic risks for asthma in the Asian population. Ten tagSNP polymorphisms for the *CHI3L1* gene were selected from the HapMap database and genotyped using a TaqMan allelic discrimination assay.

**Results:**

Adjusted odds ratios of the *CHI3L1* rs1538372 CC genotype (aOR = 1.97, 95% CI: 1.23–3.14) and the rs10399931 GG genotype (aOR = 1.77, 95% CI: 1.13–2.77) were significantly associated with asthma in the Taiwanese populations. Predictive values of forced expiratory volume in the first second of the forced vital capacity (12.37%, *P* = 0.03) and of forced vital capacity (12.10%, *P* = 0.036) decreased in conjunction with an increase in YKL-40 levels among *CHI3L1* rs1538372 CC carriers; these values were 16.1% (*P* = 0.004) and 14.5% (*P* = 0.011), respectively, among *CHI3L1* rs10399931 GG carriers. Furthermore, steroid use by asthma patients did not affect serum YKL-40 levels, but both polymorphisms had significant effects on YKL-40 levels in asthma patients who used steroids.

**Conclusions:**

Our findings suggest that the *CHI3L1* polymorphisms rs1538372 and rs10399931 can be used as genetic markers for predicting asthma risk in the Taiwanese population.

## Background

Asthma has a complicated etiology, including airway inflammation, bronchial hyper-responsiveness, and a variable degree of airway obstruction that is influenced by environmental [1] and genetic factors [2]. This disease impacts more than 300 million people worldwide and has detrimental effects on the quality of life [3].

Chitin is a widely abundant polysaccharide in nature, which triggers allergies or asthma attacks in people. It is found in fungi, as well as many arthropods, including cockroaches, house dust mites, and seafood crustaceans (such as shrimp, prawn, and crab). The mammalian 18 glycosyl hydrolase family has three members, two of which are chitinase chitotriosidase (CHIT1) and acidic mammalian chitinase (AMCase), with evolutionarily conserved glycosyl hydrolase activities that cleave chitin and consequently regulate the innate immune response [4].

The third 18 glycosyl hydrolase family member, YKL-40, is encoded by the chitinase 3-like 1 (*CHI3L1*) gene located on chromosome 1q32.1 (also called human cartilage gp-39/*HcGp-39* or breast regression protein 39/*BRP-39*) [5,6]. YKL-40 has chitin-binding activity but lacks chitinase activity and is produced by the airway epithelium, alveolar macrophages, lung fibroblasts, chondrocytes, synovial cells, breast cells, and hepatic cells. In addition, YKL-40 plays critical roles in inflammation, tissue remodeling, and fibrosis during infection, joint disease, liver fibrosis, and cancer [7]. Additionally, novel functions of YKL-40 are mediated by the Th2-inflammation pathway, which was fully demonstrated in *BRP-39* gene knockout mice and YKL-40 transgenic mice [8].

Previous cohort studies have shown that elevated levels of serum YKL-40 are significantly associated with asthmatic severity and poor lung function, because the thickness of the bronchial subepithelial basement membrane is positively correlated with YKL-40 levels [9]. A genome-wide association study of asthma indicated that the single nucleotide polymorphism (SNP) rs4950928 in the promoter region of *CHI3L1* is associated with serum levels of YKL-40 in Western European and American populations [10,11], and another study illustrated that the rs4950928 G allele is significantly associated with asthma susceptibility in a population-based study of 6514 Danish adults [12]. Moreover, rs10399805 and rs2275353 in *CHI3L1* were found be significantly associated with atopy but not asthma in a Korean population [13].

Although another study in sixty-two asthmatic patients found YKL-40 levels correlated with exacerbation of attacks [14], whether inherent and susceptible genetic variance of *CHI3L1* affects the serum YKL-40 protein levels that cause asthmatic severity in a Taiwanese population is not known. Therefore, we selected 10 tagSNPs of the *CHI3L1* gene using Tagger pairwise methods, explored the association between the *CHI3L1* gene and asthma risk, and measured decreased lung function with forced expiratory volume in the first second of the forced vital capacity (FEV1) and forced vital capacity (FVC), which are regarded as indicators of asthmatic symptom severity.

## Methods

### Study population

We enrolled adult asthmatic patients and 1:1 age-sex matched community-based controls in southern Taiwan. The asthmatic patients were diagnosed at Kaohsiung Chang Gung Memorial Hospital (CGMH) and Kaohsiung Medical University Hospital (KMUH). The 628 adult asthmatic patients had symptoms such as cough, wheezing, episodic breathlessness, and chest tightness according to the Global Initiative for Asthma guidelines and/or an increase in FEV1 of ≥12% and at least 200 mL from the prebronchodilator value based on lung function testing [15]. The 628 healthy control subjects were recruited at local health stations in the same geographic communities as our previous description [16]. In the study, all subjects with symptoms indicating lung cancer, tuberculosis, bronchitis, or pneumonia were excluded.

All participants provided informed written consent, which was reviewed and approved by the Institutional Review Boards of CGMH and KMUH (ethics approval numbers, CGMH-101-0614C and KMUH-IRB-980553).

### Ascertainment of relevant metrics

Blood samples were collected from all participants who completed a questionnaire that collected comprehensive information regarding demographics, smoking habits, parents’ asthmatic history, history of atopic disease diagnosed by a physician, steroid use (including oral or inhaled steroids), and environmental exposure factors. Spirometry was performed according to the American Thoracic Society standards [17], and FEV1and FVC were measured. All recorded data were measured three times, and the highest score was recorded under stable conditions. Total blood serum immunoglobulin E (IgE) and YKL-40 protein levels were measured using a microparticle enzyme immunoassay (IMX system Abbot, Tokyo, Japan) and the DuoSet® ELISA Development System (R&D Systems, Inc., Minneapolis, MN, USA), respectively.

### TagSNP selection and genotyping

The *CHI3L1* tagSNPs (rs903358, rs7542294, rs946259, rs880633, rs12128727, rs1538372, rs10399805, rs10399931, rs6691378, and rs946261) were selected by using the Tagger pairwise and linkage disequilibrium (LD) measurement methods on the HapMap2009 NCBI B36 assembly Chinese Han (CHB) database with minor allele frequency (MAF) ≥0.05 and *r*^2^ ≥ 0.80. Linkage disequilibrium blocks of the *CHI3L1* gene region extending from 201,413 kb to 201,430 kb on chromosome 1 were established in Haploview version 4.2 (http://www.broadinstitute.org) (Figure S1 in the online Additional file 1). Overall, the characteristics of the 10 tagSNPs are described in Table S1 in the online Additional file 2. Genomic DNA was isolated from peripheral blood using the Purgene DNA Isolation Kit (Gentra Systems, Inc., Minneapolis, MN, USA). Genotyping was performed with the TaqMan® SNP Genotyping Assay standard protocol with a 7900HT Fast Real-Time PCR System (Applied Biosystems, Foster City, CA, USA). Duplicate genotyping of 10% of the samples (selected randomly) was performed for quality control.

### Statistical analysis

All analyses were performed using IBM SPSS Statistics release 19.0 (SPSS Inc., an IBM Company, Chicago, IL, USA). Comparisons of the asthmatic and control subjects were conducted using Pearson’s chi-squared test for the dichotomous variables and the *t* test for the continuous variables. Serum IgE and YKL-40 levels did not show a normal distribution on the Shapiro–Wilk test (*P* < 0.001) and needed to be log-transformed for normality. We performed using the Pearson’s chi-squared test to assess Hardy–Weinberg equilibrium for allele distribution in the asthmatic and control groups. Adjusted odds ratios (aORs) with 95% confidence intervals (CI) according to carriers of recessive, dominant, and co-dominant genotypes were assessed with logistic regression analysis adjusting for sex, age, body mass index (BMI), log IgE, smoking, exposure to pets, indoor incense burning, and parental asthma history. To examine the effects of CHI3L1 genotype on quantitative clinical characteristics in asthmatic patients and controls groups, we performed ANOVA with a Bonferroni adjustment (post hoc comparison) and multiple linear regressions adjusting for age, sex, log IgE, BMI, and smoking as covariates.

## Results

### Characteristics of patients

Between the 628 asthmatic patients and 628 healthy controls, there were significant differences in body weight, BMI, waist circumference, hip circumference, waist-to-hip ratio, serum IgE and YKL-40 levels, lung function, smoking and drinking behavior, exposure to indoor pets and incense burning, and parents’ asthma history (Table 1).

**Table 1:** The demographic characteristics of asthmatics and controls

	**Asthma N**^**a**^ **= 628**	**Control N**^**a**^ **= 628**	***P *** **value**^**d**^
Age (years)^b^	51.44 ± 14.13	50.42 ± 11.84	0.522
Sex			
Male	289 (46.0%)	289 (46.0%)	1.000
Female	339 (54.0%)	339 (54.0%)	
Height (cm)^b^	162.15 ± 8.33	161.82 ± 8.28	0.481
Weight (kg)^b^	66.56 ± 12.47	63.04 ± 11.86	<0.001
BMI (kg/m^2^)^ab^	25.27 ± 4.07	23.95 ± 3.40	<0.001
WC (cm)^ab^	86.93 ± 11.18	78.58 ± 9.51	<0.001
Hips (cm)^b^	99.33 ± 8.88	95.74 ± 6.62	<0.001
WHR^ab^	0.88 ± 0.08	0.82 ± 0.08	<0.001
IgE (IU/mL)^b^	262.04 ± 584.65	128.07 ± 291.48	<0.001^c^
YKL-40 (ng/mL)^b^	112.84 ± 111.56	95.65 ± 101.46	0.005^c^
Lung function^b^			
FEV1%, pred	81.75 ± 21.53	92.17 ± 16.41	<0.001
FVC%, pred	85.75 ± 21.31	97.36 ± 17.05	<0.001
FEV1/FVC ratio	0.96 ± 0.15	0.95 ± 0.12	0.843
Smokers			
Current	69 (11.1%)	82 (13.1%)	<0.001
Past	99 (15.9%)	44 (7.0%)	
Never	456 (73.1%)	502 (79.9%)	
Drinking habits			
Current	72 (11.5%)	60 (9.7%)	0.012
Past	38 (6.1%)	18 (2.9%)	
Never	515 (82.4%)	541 (87.4%)	
Indoor environmental factors			
Pets (dogs, cats, birds)	190 (30.4%)	103 (21.5%)	0.001
Appearance of cockroaches	491 (%)	(%)	
Air cleaner	102 (16.3%)	66 (13.8%)	0.245
Dehumidifiers	140 (22.5%)	111(23.4%)	0.737
Carpet	26 (4.2%)	18 (3.8%)	0.752
Mold in walls	108(17.3%)	98(20.6%)	0.171
Indoor incense burning	402 (64.5%)	215 (45.2%)	<0.001
Asthma history for father, yes	69 (11.2%)	23 (4.6%)	<0.001
Asthma history for mother, yes	51 (8.2%)	17 (3.4%)	<0.001

^a^*Abbreviations:BMI* body mass index, *WC* waist circumference, *WHR* waist-to-hips ratio, *N* number of individuals.

^b^Values are expressed as the means ± standard deviation (SD) unless otherwise stated.

^c^The *P* value of the IgE and YKL-40 serum levels was determined by *t* test after log-transformation.

^d^The *P* value was calculated for the continuous variables by the *t* test, and the χ^2^ test was used for the categorical variable.

### C*HI3L1* tagSNP association study

For the association analysis, we identified 10 tagSNPs for *CHI3L1* from the HapMap 2009 NCBI B36 assembly of the Chinese Han database (Table S1 in Additional file 2). The association results are presented in Table S2 in Additional file 3. *CHI3L1* genotypes and allele carrier frequencies were calculated in the asthmatic patients and controls.

No deviation from Hardy–Weinberg equilibrium was observed for the 10 tagSNPs. Only rs1538372 (*P* = 0.014) and rs10399931 (*P* = 0.047) had a nominally significant association with asthma, respectively. However, the two polymorphisms rs1538372 (*P* = 0.14) and rs10399931 (*P* = 0.47) and other eight genotypes were not significantly associated with asthma after applying the Bonferroni correction.

Furthermore, we observed that for the aOR in rs1538372, the CC genotype was associated with an increased risk of asthma compared with the TT genotype (aOR = 1.97, 95% CI 1.23–3.14). For rs10399931, the GG genotype was associated with an increased risk of asthma compared with the AA genotype (aOR = 1.77, 95% CI 1.13–2.77) (Table 2).

**Table 2:** **The adjusted odd ratios between asthma status and the *CHI3L1 *** **polymorphisms rs1538372 and rs10399931 according to three inheritance modes**

**Categories**	**Case/Control**	**OR (95% CI)**	**aOR (95% CI)**^**a**^
rs1538372			
Co-dominant genotype			
TT	65/90	1	1
TC	293/312	1.3 (0.91–1.86)	1.54 (0.98–2.43)
CC	270/226	1.65 (1.15–2.38)	1.97 (1.23–3.14)
Recessive genotype			
TT + TC	358/402	1	1
CC	270/226	1.34 (1.07–1.68)	1.39 (1.04–1.86)
Dominant genotype			
TT	65/90	1	1
TC + CC	563/538	1.45 (1.03–2.04)	1.72 (1.11–2.66)
rs10399931			
Co-dominant genotype			
AA	72/90	1	1
GA	286/307	1.16 (0.82–1.65)	1.36 (0.88–2.10)
GG	270/229	1.47 (1.03–2.11)	1.77 (1.13–2.77)
Recessive genotype			
AA + GA	358/397	1	1
GG	270/229	1.31 (1.04–1.64)	1.39 (1.04–1.86)
Dominant genotype			
AA	72/90	1	1
GA + GG	556/536	1.30 (0.93–1.81)	1.53 (1.01–2.32)

^a^Adjusted odds ratio and 95% confidence intervals (aOR and 95% CI) are shown using multiple logistic regressions to control variables, including sex, age, BMI, logIgE, smoking, pet exposure, indoor incense burning, and parents’ asthma history.

### Association of clinical quantitative characteristics

We investigated the differences in clinical quantitative traits, including adiposity, serum levels of IgE and YKL-40, and lung function, among subjects with *CHI3L1* rs1538372 and rs10399931 genotypes in asthmatic patients and control groups. A significant association was revealed between the rs1538372 and rs10399931 genotypes and serum YKL-40 protein levels (Table 3). In this study, we found that FEV1% and FVC% decreased by 12.37% (*P* = 0.03) and 12.10% (*P* = 0.036), respectively, as YKL-40 levels were higher in *CHI3L1* rs1538372 CC carriers than in TC or TT carriers. Similarly, FEV1% and FVC% in subjects with *CHI3L1* rs10399931 GG genotype were significantly lower at -16.1% (*P* = 0.004) and -14.5% (*P* = 0.011), respectively (Table 4).

**Table 3:** **The associations of the *CHI3L1 *** **rs1538372 and rs10399931 polymorphisms with clinical status and characteristics in asthma and control groups**

	**Asthma**				**Control**			
**rs1538372**	**CC (N = 270)**	**CT (N = 293)**	**TT (N = 65)**	***P *** **value**	**CC (N = 226)**	**CT (N = 311)**	**TT (N = 91)**	***P *** **value**
Age (year)	51.40 ± 14.26	51.14 ± 14.08	52.91 ± 13.91	0.66	50.00 ± 11.86	50.78 ± 11.64	50.22 ± 12.58	0.743
Sex								
Male	120 (44.4%)	142 (48.5%)	27 (41.5%)	0.472	108 (47.8%)	141 (45.3%)	40 (44.0%)	0.779
Female	150 (55.6%)	151(51.5%)	38 (58.5%)		118 (52.2%)	170 (54.7%)	51 (56.0%)	
BMI (kg/m^2^)	25.31 ± 4.25	25.3 ± 3.93	25.00 ± 3.92	0.846	23.85 ± 3.54	23.88 ± 3.31	24.47 ± 3.36	0.332
WC (cm)	86.70 ± 12.00	87.18 ± 10.83	86.74 ± 8.89	0.888	79.21 ± 10.20	77.67 ± 9.03	80.06 ± 9.02	0.113
WHR	0.88 ± 0.09	0.88 ± 0.08	0.87 ± 0.06	0.764	0.83 ± 0.10	0.81 ± 0.07	0.82 ± 0.07	0.041
IgE (IU/mL)	279.24 ± 648.79	221.09 ± 399.22	375.48 ± 913.44	0.7^a^	111.64 ± 190.17	137.02 ± 337.76	140.32 ± 336.82	0.619^a^
YKL-40 (ng/mL)	130.00 ± 126.89	103.79 ± 101.13	81.18 ± 69.18	< 0.0001^a^	115.47 ± 128.54	88.441 ± 84.23	68.32 ± 61.35	0.011^a^
FEV1%, pred	84.00 ± 20.66	79.71 ± 21.73	79.26 ± 23.42	0.046	91.91 ± 17.46	93.36 ± 15.64	88.75 ± 15.88	0.16
FVC%, pred	87.47 ± 21.03	84.32 ± 20.40	84.94 ± 25.67	0.216	97.08 ± 16.90	97.52 ± 17.78	97.54 ± 15.01	0.97
**rs10399931**	**GG (N = 270)**	**GA (N = 286)**	**AA (N = 72)**	***P *** **value**	**GG (N = 229)**	**GA (N = 307)**	**AA (N = 90)**	***P *** **value**
Age (year)	51.28 ± 14.14	51.16 ± 14.06	53.13 ± 14.41	0.557	50.32 ± 11.63	50.32 ± 11.79	50.97 ± 12.72	0.892
Sex								
Male	122 (45.2%)	136 (47.6%)	31 (43.1%)	0.741	112 (48.9%)	136 (44.3%)	40 (44.4%)	0.542
Female	148 (54.8%)	150 (52.4%)	41 (56.9%)		117 (51.1%)	171 (55.7%)	50 (55.6%)	
BMI (kg/m^2^)	25.24 ± 4.29	25.36 ± 3.87	25.06 ± 4.04	0.843	23.82 ± 3.33	23.96 ± 3.46	24.16 ± 3.34	0.723
WC (cm)	86.54 ± 11.91	87.08 ± 10.87	87.78 ± 9.47	0.712	78.84 ± 9.71	78.08 ± 9.50	79.40 ± 9.01	0.534
WHR	0.87 ± 0.09	0.88 ± 0.08	0.88 ± 0.07	0.942	0.83 ± 0.10	0.82 ± 0.07	0.82 ± 0.07	0.399
IgE (IU/mL)	276.80 ± 645.41	229.82 ± 454.97	331.63 ± 768.03	0.613	95.65 ± 155.72	150.11 ± 346.92	139.44 ± 351.86	0.511
YKL-40 (ng/mL)	133.08 ± 128.56	99.16 ± 89.75	90.66 ± 109.85	< 0.0001^a^	105.15 ± 103.82	98.89 ± 108.91	63.403 ± 57.64	0.009^a^
FEV1%, pred	83.20 ± 20.89	80.96 ± 22.16	77.41 ± 21.05	0.116	92.81 ± 18.37	92.91 ± 14.58	88.30 ± 16.71	0.127
FVC%, pred	86.79 ± 20.83	85.09 ± 21.67	84.44 ± 21.79	0.562	98.69 ± 18.26	96.42 ± 16.31	97.18 ± 16.48	0.463

^a^*P* value was presented by testing the total serum IgE and YKL-40 levels after log-transformation to follow normal distribution. (ANOVA and Bonferroni).

**Table 4:** **The correlations between serum YKL-40 levels and lung functions according to the *CHI3L1 *** **polymorphisms in asthmatics**

	**Lung function FEV1% predicted**			**Lung function FVC% predicted**		
**Serum YKL40 level (log-transformation)**	**β ± SE**	***r *** **coefficient**	***P *** **value**^**a**^	**β ± SE**	***r *** **coefficient**	***P *** **value**^**a**^
rs1538372						
TT	-3.61 ± 11.768	-0.049	0.761	0.379 ± 13.74	0.004	0.978
TC	-11.38 ± 5.20	-0.151	0.03	-12.95 ± 5.01	-0.177	0.01
CC	-12.37 ± 5.526	-0.158	0.026	-12.10 ± 5.71	-0.149	0.036
rs10399931						
AA	-12.11 ± 8.644	-0.202	0.168	-11.60 ± 9.95	-0.17	0.249
GA	-6.90 ± 5.83	-0.083	0.238	-8.98 ± 5.74	-0.11	0.119
GG	-16.10 ± 5.50	-0.205	0.004	-14.5 ± 5.70	-0.18	0.011

^a^Linear regression was performed adjusting for age, sex, logIgE, BMI, and smoking.

**Figure 1:**
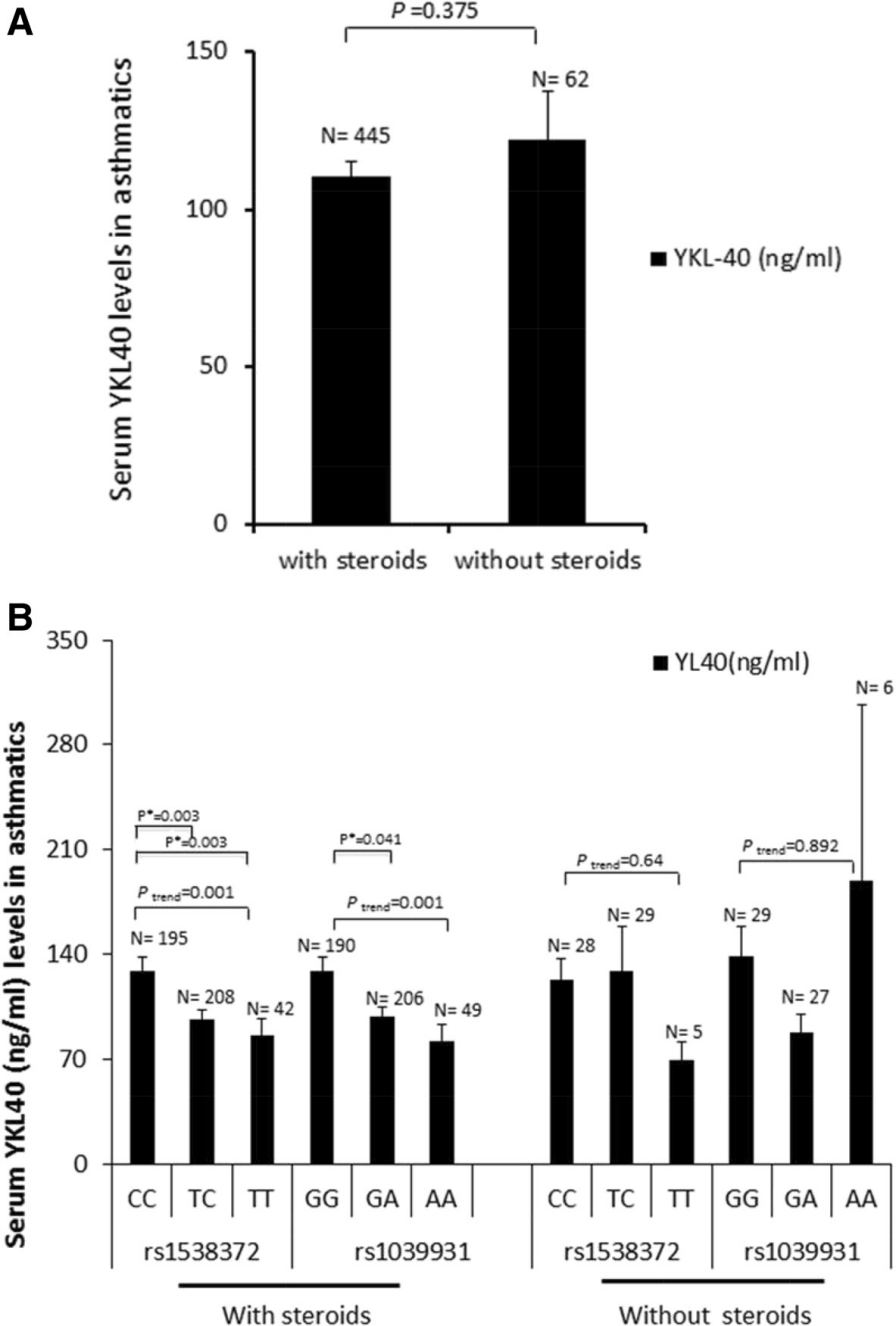
**The YKL-40 levels were affected by rs1538372 and rs10399931 genotypes in asthmatics. (A)** The serum YKL-40 levels were assessed in asthmatics using steroids or not using steroids. Mean ± standard error (SE) is shown with significance assessed by *t* test. **(B)** The serum YKL-40 levels were analyzed according to rs1538372 and rs10399931 genotypes in asthmatics using steroids or not using steroids. Mean ± standard error (SE) is shown. The mean trend tests were performed by entering cross-categorized variables as a continuous variable into the linear regression model and including multiple testing corrections (Bonferroni; P* < 0.05).

Further, using *CHI3L1* polymorphisms, we determined whether steroid use affected serum YKL-40 levels in asthmatics. We found no significant difference in serum YKL-40 levels between asthmatic subjects who did and did not use steroids (Figure 1A). However, it is noteworthy that YKL-40 levels varied by rs1538372 genotypes (CC > TC > TT, *P* for trend = 0.001) and rs10399931 (GG > GA > AA, *p* for trend = 0.001) in asthmatic patients who used steroids (Figure 1B).

## Discussion

Following the discovery that *CHI3L1* variation affects serum YKL-40 levels, the risk of asthma and impaired lung function were studied in Western European and American populations with a genome-wide association method [10]. In fact, we are the first to demonstrate that the *CHI3L1* polymorphisms rs1538372 and rs10399931 in the Taiwanese population were not only significantly associated with asthma risk, but also related to serum YKL-40 concentrations with a gene-dose dependent relationship. At the same time, we found that the percent predictive value of FEV1 and FVC among *CHI3L1* rs1538372 CC carriers was lower than in TC or TT carriers, and similar findings were observed for rs10399931 GG carriers compared to GA or AA carriers.

In a Korean population study (198 asthmatics and 277 non-asthmatics), the C versus T allele of the rs1538372 polymorphism and the G versus A allele of rs10399931 polymorphism were associated with an increased risk of asthma (OR = 1.26 and 1.19, respectively) [13]. However, the differences were not statistically significant; therefore, it was concluded that the two polymorphisms did not increase asthma risk. Interestingly, our matched data set (628 asthmatics and 628 controls) gave significant odds ratios for the rs1538372 C allele (OR = 1.27, 95% CI: 1.08-1.49) and the rs10399931 G allele (OR = 1.22, 95% CI: 1.04-1.44) associations with asthma. The differences between the studies are due to the different sample sizes. Overall, the two SNPs were consistently associated with increased asthma risk and could be considered important risk markers for asthma in Asians.

Verlann and coworkers [18] constructed luciferase-based reporter vectors including three variant genotypes: the rs10399931 C allele (complementary to the G risk allele on the reverse strand), the rs10399805 A allele, and the rs4950928 C allele. Their study found that the haplotype CAC (or GTG haplotype) has the highest promoter activity compared with the other haplotypes. They concluded that rs103999331 and rs4950925 are equally likely to modulate *CHI3L1* expression and susceptibility to asthma in Caucasian and African populations.

To treat asthmatic patients in the clinical setting, inhaled or oral glucocorticoids are commonly administered, owing to their direct inhibitory effects on airway inflammation [19]. Glucocorticoid is a type of steroid hormone that binds to the glucocorticoid receptor to translocate into the nucleus, where it binds to specific DNA elements to regulate gene expression [20]. We found that the transcription factor–binding sites of glucocorticoid receptor element (GRE) and STAT1 are closer to rs1538372 and that cAMP response element–binding protein (CREBP1) and E4 promoter–binding protein 4 (E4BP4) are closer to rs10399931 from UCSC Genome Browser (NCBI36/hg18) [21,22] (Figure S2 in the online Additional file 4 and Table S3 in the online Additional file 5). There is convincing evidence for STAT1 constitutive activation in the asthmatic airway epithelium [23]. The E4BP4 protein, which binds to elements located on the promoters of the repressed COX-2 and iNOS genes, was induced by glucocorticoid dexamethasone [24]. Thus, the two SNPs in the *CHI3L1* gene might be affected by glucocorticoids.

Here, we first found that serum YLK-40 levels were not different between asthmatic patients who did or did not use steroids. Furthermore, serum YKL-40 levels were highly elevated by dependent genotypes in steroid-using asthma patients with rs1538372 CC and rs10399931 GG. These results showed that the rs1538372 CC and rs10399931 GG are risk genotypes associated with severity of asthmatic lung function. The steroid-using asthmatic subjects who have genotypes rs1538372 CC and rs10399931 GG will be examined in a future study to clarify whether these risk carriers might be associated with steroid resistance.

Here, we summarized the mechanisms of regulation of *CHI3L1* gene expression on bronchial epithelium, smooth muscle, monocytes, and macrophages to elucidate the role of the *CHI3L1* gene in asthmatic pathology. We factored in numerous environmental factors, such as smoking, house dust mite exposure [25], rhinovirus [26], lipopolysaccharide infection [27], and interleukin 13 [8]. It is well known that the AKT-MAPK-ATF2 or STAT1 signaling pathways are implicated in YKL-40 expression or exacerbation of asthma symptoms (Figure S3 in the online Additional file 6).

Overall, the limitations of the study need to be discussed. We found that the *CHI3L1* polymorphisms rs1538372 and rs10399931 had an *r*^2^ of 0.84 in a linkage disequilibrium plot (Additional file 2: Table S1) and a nominally significant association with asthma (*P* < 0.05). The correlations were not significant after Bonferroni correction. However, two SNPs were significantly associated with asthma and significantly associated with serum YKL-40 levels and lung functions in asthmatic according to multiple regression models after adjusting for potential confounding factors. Importantly, we managed to reduce false positive results and avoid excluding small effects through the overly conservative Bonferroni correction. Furthermore, there are still limitations that require further large-scale exploration to clarify gene-gene and gene-environment interactions.

Taken together, protein YKL-40 levels are elevated in many kinds of diseases, such as asthma, multiple sclerosis, rheumatoid arthritis, osteoarthritis, infectious diseases, cardiovascular disease, and cancer. Thus, serum YKL-40 levels are not a specific diagnosis for one disease, and now several studies question whether unregulated YKL-40 is a good diagnostic biomarker in many diseases. YKL-40 may not be a good biomarker, but it can reflect poor prognosis [7] and overall mortality risk [28].

## Conclusion

Our findings suggest that *CHI3L1* polymorphisms rs1538372 and rs10399931 can be used as genetic markers predicting asthma risk in the Taiwanese population.

## Competing interests

All authors declare that they have no competing financial or personal interests.

## Authors’ contributions

WT conceived and designed the research. HM, LM, WC, and WC collected clinical samples. CY, LJ and TY performed the *CHI3L1* genotyping and ELISA assays. WT, CY, and TS participated in statistical analysis. TS drafted the manuscript and KY edited and reviewed the manuscript. All authors approved the final version of the manuscript.

## Pre-publication history

The pre-publication history for this paper can be accessed here:

http://www.biomedcentral.com/1471-2350/15/86/prepub

## Supplementary Material

Additional file 1: Figure S1 Linkage disequilibrium plot based on the HapMap CHB database. The number in each square represents the standard color scheme (*r*^2^) between each pair of SNPs. Ten SNPs (rs903358, rs7542294, rs946259, rs880633, rs12128727, rs1538372, rs10399805, rs10399931, rs6691378, and rs946261) are shown. Click here for file

Additional file 2: Table S1 The ten Tag SNPs of the *CHI3L1* primers for TaqMan PCR in this study. Click here for file

Additional file 3: Table S2 The associations between asthma and ten *CHI3L1* tag SNPs. Click here for file

Additional file 4: Figure S2 Box1 (CREBP1 and E4BP4 with rs4950928 and rs10399931) and Box2 (GRE and STAT1 with rs1538372) show the conserved transcription factor binding sites (■) and the positions corresponding to the single nucleotide polymorphism (I) in different ethnic groups, predicted on chromosome 1 and positioned from 201,420,794 to 201,422,994 (total 2201 bp) by the UCSC Genome Browser (NCBI36/hg18) assembly. Click here for file

Additional file 5: Table S3 The features of the transcription factor binding sites in the *CHI3L1* promoter region. Click here for file

Additional file 6: Figure S3 The mechanisms of regulation of *CHI3L1* gene expression on bronchial epithelium, smooth muscle, monocytes, and macrophages by environmental factors. The solid line indicates that the mechanisms have direct interactions from the journal articles (# References number) and the dotted line indicates proposed but unverified pathways. ATF2, activating transcription factor 2; PI3K, phosphoinositide-3-kinase; AKT, serine–threonine protein kinase. ERK, extracellular signal-regulated kinase; IL13, interleukin 13; IFN-γ, interferon gamma; LPS, lipopolysaccharide. C/EBP-alpha, CCAAT/Enhancer Binding Protein, Alpha. Click here for file
