# Supplementary material for: CHI3L1 polymorphisms associate with asthma in a Taiwanese population
Source: BMC Med Genet. 2014 Jul 23;15:86. doi: 10.1186/1471-2350-15-86 (PMC4113488; doi:10.1186/1471-2350-15-86)
Supplement: Additional file 1: Figure S1 — Linkage disequilibrium plot based on the HapMap CHB database. The number in each square represents the standard color scheme (r2) between each pair of SNPs. Ten SNPs (rs903358, rs7542294, rs946259, rs880633, rs12128727, rs1538372, rs10399805, rs10399931, rs6691378, and rs946261) are shown. [file 1471-2350-15-86-S1.pdf]

Figure S1

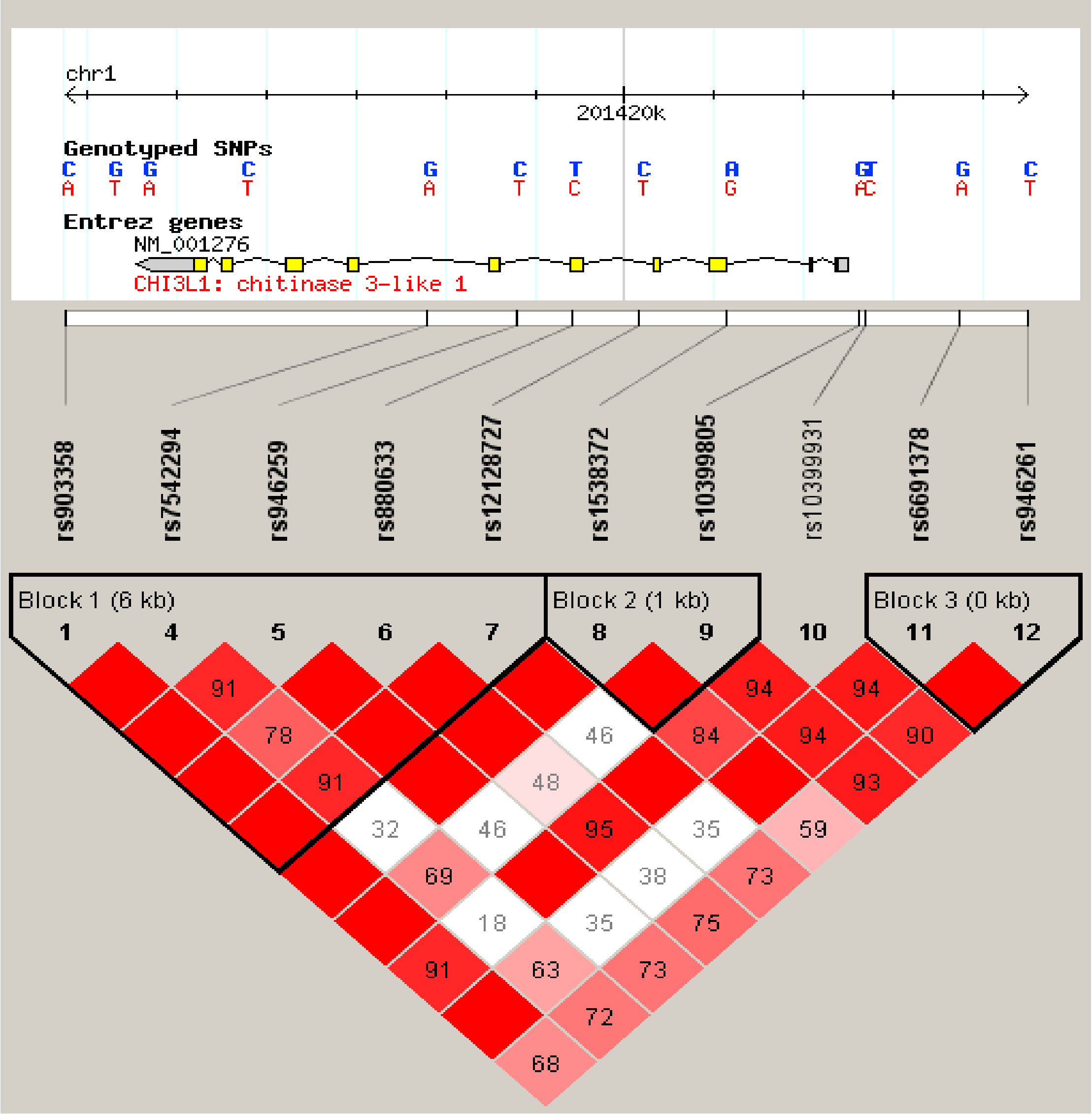
chr1
201420k
Genotyped SNPs
Entrez genes
NM_001276
CHI3L1: chitinase 3-like 1
rs903358
rs7542294
rs946259
rs880633
rs12128727
rs1538372
rs10399805
rs10399931
rs6691378
rs946261
Block 1 (6 kb)
Block 2 (1 kb)
Block 3 (0 kb)
1 4 5 6 7 8 9 10 11 12
