# Supplementary material for: CHI3L1 polymorphisms associate with asthma in a Taiwanese population
Source: BMC Med Genet. 2014 Jul 23;15:86. doi: 10.1186/1471-2350-15-86 (PMC4113488; doi:10.1186/1471-2350-15-86)
Supplement: Additional file 2: Table S1 — The ten Tag SNPs of the CHI3L1 primers for TaqMan PCR in this study. [file 1471-2350-15-86-S2.pdf]

Table S1. The ten Tag SNPs of the *CHI3L1* primers for TaqMan PCR in this study

	Tag-SNPs	Context Sequence [VIC/FAM]	Function class	Position (NCBI36/hg18)	Position (GRCh37)
Block1	rs903358	CCTCCTTCCCTCCACTTCAAACCGG[A/C] GTCACAAACCATCAGGCCAATTTGT		201413762	203147139
	rs7542294	GTGGATTGCATGAATACGGGTATGT[A/G] GGGCAAATGGTTGACATCCACCTGT	intron	201417799	203151176
	rs946259	TCCTTTTCCTAAAAAACTGACTAAT[C/T] TTCTCCCCAAACCCATGCACACCCA	intron	201418800	203152177
	rs880633	AGGGTGGTAAAATGCTGTTTGTCTC[C/T] CCGTCCAGGGTAGAGCCAGGCAAGG	Exon 5	201419424	203152801
	rs12128727	TGGGCTCTGCTTTCTTTGCTACA[C/T]AT CAGGCAGGCCCTTGACAAACAGG	intron	201420178	203153555
Block2	rs1538372	GCAGAGCCTGAAGGAGAAGTCTGGG[A/ G]TGGGGCCCGGGCCAGGATTCGGCAA	intron	201421155	203154532
	rs10399805	ATTACCAGAGGAGGGTTGAGAAACC[A/ G]CAGAGTTTTGAAAACTTTGGGTCAG	nearGene5’	201422621	203155998
	rs10399931	CACTAGGGTGATGATGGGGGGCTTC[C/T] GGAGATGTGACTCAGCCGCATTTCC	nearGene5’	201422703	203156080
Block3	rs6691378	AAGTGGCTTGTCCAGAATCACGCTC[A/G]GTGAATACTAAAGAGGCATCACTTT	nearGene5’	201423745	203157122
	rs946261	CTCACAAGGGCAGTGTTGGAGGAGC[C/ T]CAGGTAGATGCCATGAATGCAATGA	nearGene5’	201424496	203157873
