# Supplementary material for: CHI3L1 polymorphisms associate with asthma in a Taiwanese population
Source: BMC Med Genet. 2014 Jul 23;15:86. doi: 10.1186/1471-2350-15-86 (PMC4113488; doi:10.1186/1471-2350-15-86)
Supplement: Additional file 3: Table S2 — The associations between asthma and ten CHI3L1 tag SNPs. [file 1471-2350-15-86-S3.pdf]

Table S2. The associations between asthma and ten *CHI3L1* tag SNPs

		Asthma (N=628)	Control (N=628)	*P* value	Allele Frequency and Hardy-Weinberg equilibrium *P* value [a]		
					Type	Asthma	control
rs903358	GG	24 (3.8%)	24 (3.8%)	0.568	G	18.96%	20.33%
	TG	189 (30.2%)	207 (30.3%)		T	81.04%	79.67%
	TT	412 (65.9%)	396 (63.2%)			*P*= 0.6899	*P*= 0.6347
rs7542294	TT	115 (18.3%)	104 (16.6%)	0.577	T	42.6%	42.2%
	TC	305 (48.6%)	322 (51.3%)		C	57.4%	57.8%
	CC	208 (33.1%)	202 (32.2%)			*P*= 0.8631	*P*= 0.2006
rs946259	AA	81 (12.9%)	65 (10.4%)	0.368	A	34.95%	32.88%
	GA	277 (44.1%)	283 (45.1%)		G	65.05%	67.12%
	GG	270 (43.0%)	280 (44.6%)			*P*= 0.4525	*P*= 0.5998
rs880633	GG	90 (14.4%)	72 (11.5%)	0.309	G	37.56%	35.27%
	GA	291(46.4%)	299 (47.6%)		A	62.44%	64.73%
	AA	246 (39.2%)	257(40.9%)			*P*= 0.7923	*P*= 0.2844
rs12128727	AA	79 (12.6%)	65 (10.4%)	0.444	A	34.72%	32.88%
	GA	276 (44.2%)	283(45.1%)		G	65.28%	67.12%
	GG	270 (43.2%)	280 (44.6%)			*P*= 0.5186	*P*= 0.5998
rs1538372	CC	270(43.0%)	226 (36.0%)	0.014	C	66.32%	60.83%
	TC	293(46.7%)	312 (49.7%)		T	33.68%	39.17%
	TT	65 (10.4%)	90 (14.3%)			*P*= 0.2657	*P*= 0.2866
rs10399805	TT	63 (10.1%)	49(7.8%)	0.083	T	30.52%	26.87%
	TC	261 (41.8%)	239(38.1%)		C	69.48%	73.13%
	CC	301 (48.2%)	339(54.1%)			*P*= 0.4602	*P*= 0.45
rs10399931	GG	270 (43.0%)	229 (36.6%)	0.047	G	65.76%	61.1%
	GA	286 (45.5%)	307 (49%)		A	34.24%	38.9%
	AA	72 (11.5%)	90 (14.4%)			*P*= 0.7758	*P*= 0.4277
rs6691378	TT	69 (11.1%)	53 (8.5%)	0.08	T	32.83%	28.64%
	TC	271(43.5%)	252 (40.3%)		C	67.17%	71.36%
	CC	283(45.4%)	320 (51.2%)			*P*= 0.7336	*P*= 0.7343
rs946261	AA	73 (11.6%)	63 (10.1%)	0.474	A	33.12%	33.07%
	GA	270 (43.0%)	288 (46.0%)		G	66.88%	66.93%
	GG	285 (45.4%)	275 (43.9%)			*P*= 0.4592	*P*= 0.3251

a.Hardy-Weinberg equilibrium was tested by Pearson’s Chi-square test
