# Supplementary figures and images for: CHI3L1 polymorphisms associate with asthma in a Taiwanese population
Source: BMC Med Genet. 2014 Jul 23;15:86. doi: 10.1186/1471-2350-15-86 (PMC4113488; doi:10.1186/1471-2350-15-86)

Figure S2

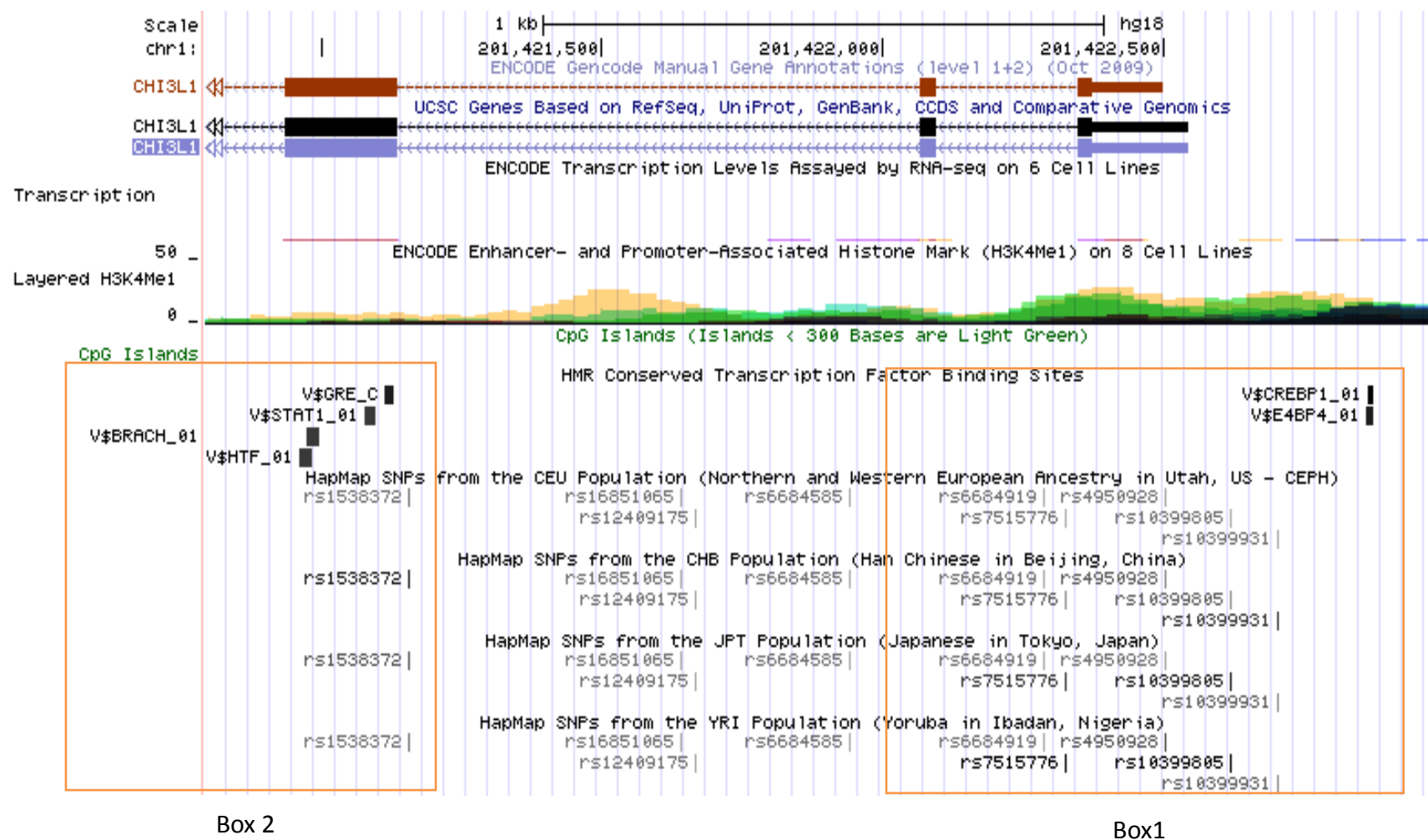

Supplement: Additional file 4: Figure S2 — Box1 (CREBP1 and E4BP4 with rs4950928 and rs10399931) and Box2 (GRE and STAT1 with rs1538372) show the conserved transcription factor binding sites (■) and the positions corresponding to the single nucleotide polymorphism (I) in different ethnic groups, predicted on chromosome 1 and positioned from 201,420,794 to 201,422,994 (total 2201 bp) by the UCSC Genome Browser (NCBI36/hg18) assembly. [file 1471-2350-15-86-S4.pdf]

Figure S3

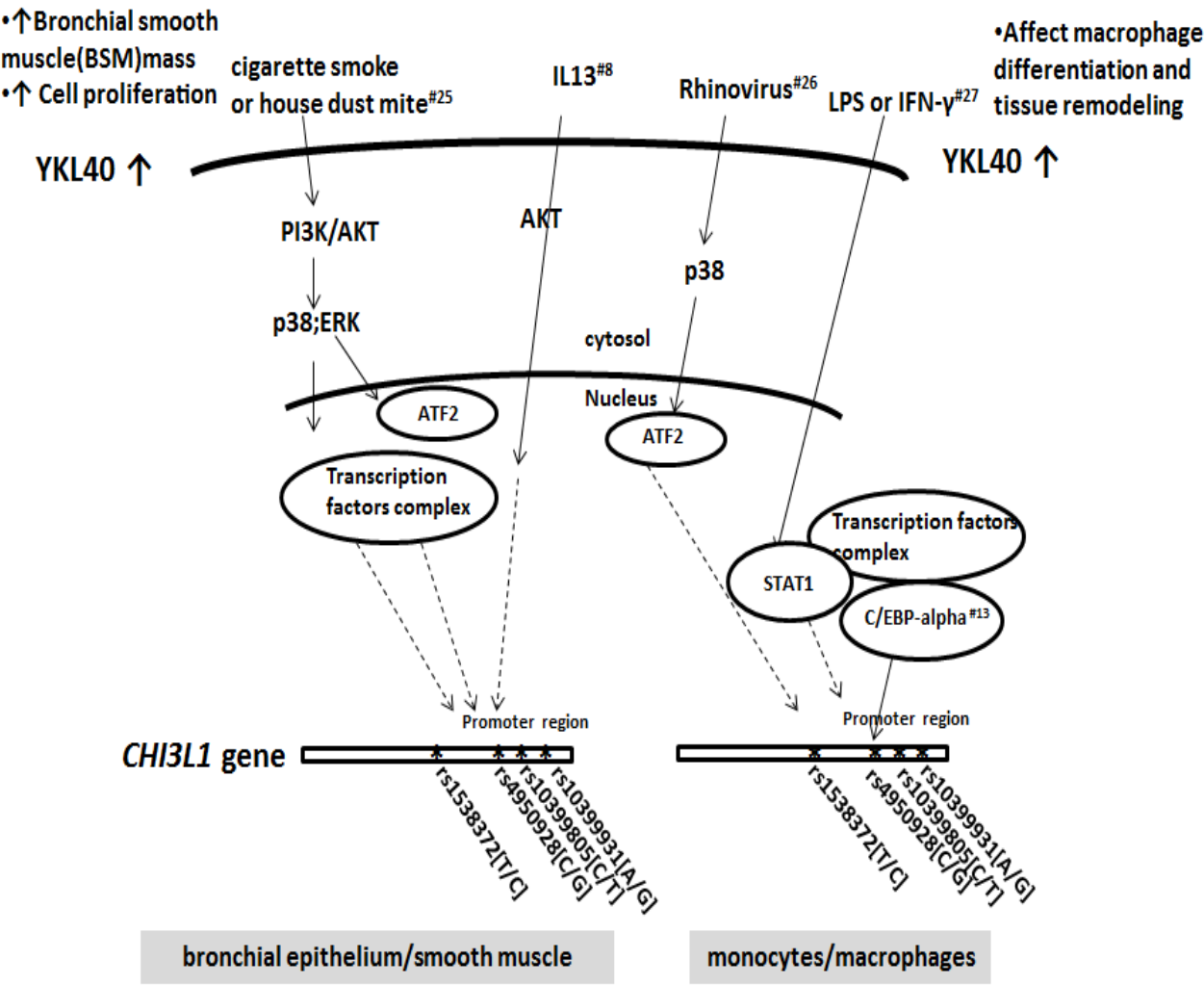

Supplement: Additional file 6: Figure S3 — The mechanisms of regulation of CHI3L1 gene expression on bronchial epithelium, smooth muscle, monocytes, and macrophages by environmental factors. The solid line indicates that the mechanisms have direct interactions from the journal articles (# References number) and the dotted line indicates proposed but unverified pathways. ATF2, activating transcription factor 2; PI3K, phosphoinositide-3-kinase; AKT, serine–threonine protein kinase. ERK, extracellular signal-regulated kinase; IL13, interleukin 13; IFN-γ, interferon gamma; LPS, lipopolysaccharide. C/EBP-alpha, CCAAT/Enhancer Binding Protein, Alpha. [file 1471-2350-15-86-S6.pdf]
