# Supplementary material for: CHI3L1 polymorphisms associate with asthma in a Taiwanese population
Source: BMC Med Genet. 2014 Jul 23;15:86. doi: 10.1186/1471-2350-15-86 (PMC4113488; doi:10.1186/1471-2350-15-86)
Supplement: Additional file 5: Table S3 — The features of the transcription factor binding sites in the CHI3L1 promoter region. [file 1471-2350-15-86-S5.pdf]

Table S3. The features of the transcription factor binding sites in the *CHI3L1* promoter region

Transcription factor and Synonyms		Swissprot ID	Entrez Gene:	Binding Site Position in *CHI3L1*		Span size (nt)	Distance from the binging site to the related SNP position	
							rs1538372 [a]	rs10399931[b]
GR	Glucocorticoid receptor; ***NR3C1***	P04150	2908	201421115	201421130	16	-40 ~ -25	-1588~1573
STAT1	*STAT1*	P42224	6772	201421078	201421098	21	-77 ~ -57	-1625~-1605
BRACH	Brachyury Protein; ***T***	P20293	6862	201420975	201420998	24	-180~ -157	-1728~1705
HTF	Hepatocarcinogenesis-related transcription factor; ***XBP1***	Q9R1S4	7494	201420961	201420984	24	-194~ -171	-1742~-1719
E4BP4	E4 promoter-binding protein 4; ***NFIL3***	Q16649	4783	201422864	201422875	12	+1709~+1720	+161~+172
CREBP1	cAMP response element-binding protein CRE-BP1; ***ATF2***	P15336	1386	201422866	201422873	8	+1711~ +1718	+163~+170

The distance from the transcription factor binding site to the SNP site were related to the SNP (rs1538372 or rs10399931) position and orientation with "+" for up-stream and "-" for down-stream, relating to transcription start site position 201422500. The data were analysed using the UCSC genome browser assembly mapping 2006 (NCBI36/hg18).

a. The SNP rs1538372 position is 201421155.

b. The SNP rs10399931 position is 201422703.
